# A Phasin with Many Faces: Structural Insights on PhaP from *Azotobacter* sp. FA8

**DOI:** 10.1371/journal.pone.0103012

**Published:** 2014-07-31

**Authors:** Mariela P. Mezzina, Diana E. Wetzler, Mariela V. Catone, Hernan Bucci, Matias Di Paola, M. Julia Pettinari

**Affiliations:** Departamento de Química Biológica, Facultad de Ciencias Exactas y Naturales, Universidad de Buenos Aires, IQUIBICEN-CONICET, Buenos Aires, Argentina; Centro Nacional de Biotecnologia - CSIC, Spain

## Abstract

Phasins are a group of proteins associated to granules of polyhydroxyalkanoates (PHAs). Apart from their structural role as part of the PHA granule cover, different structural and regulatory functions have been found associated to many of them, and several biotechnological applications have been developed using phasin protein fusions. Despite their remarkable functional diversity, the structure of these proteins has not been analyzed except in very few studies. PhaP from *Azotobacter* sp. FA8 (PhaP_Az_) is a representative of the prevailing type in the multifunctional phasin protein family. Previous work performed in our laboratory using this protein have demonstrated that it has some very peculiar characteristics, such as its stress protecting effects in recombinant *Escherichia coli*, both in the presence and absence of PHA. The aim of the present work was to perform a structural characterization of this protein, to shed light on its properties. Its aminoacid composition revealed that it lacks clear hydrophobic domains, a characteristic that appears to be common to most phasins, despite their lipid granule binding capacity. The secondary structure of this protein, consisting of α-helices and disordered regions, has a remarkable capacity to change according to its environment. Several experimental data support that it is a tetramer, probably due to interactions between coiled-coil regions. These structural features have also been detected in other phasins, and may be related to their functional diversity.

## Introduction

**Figure 1:**
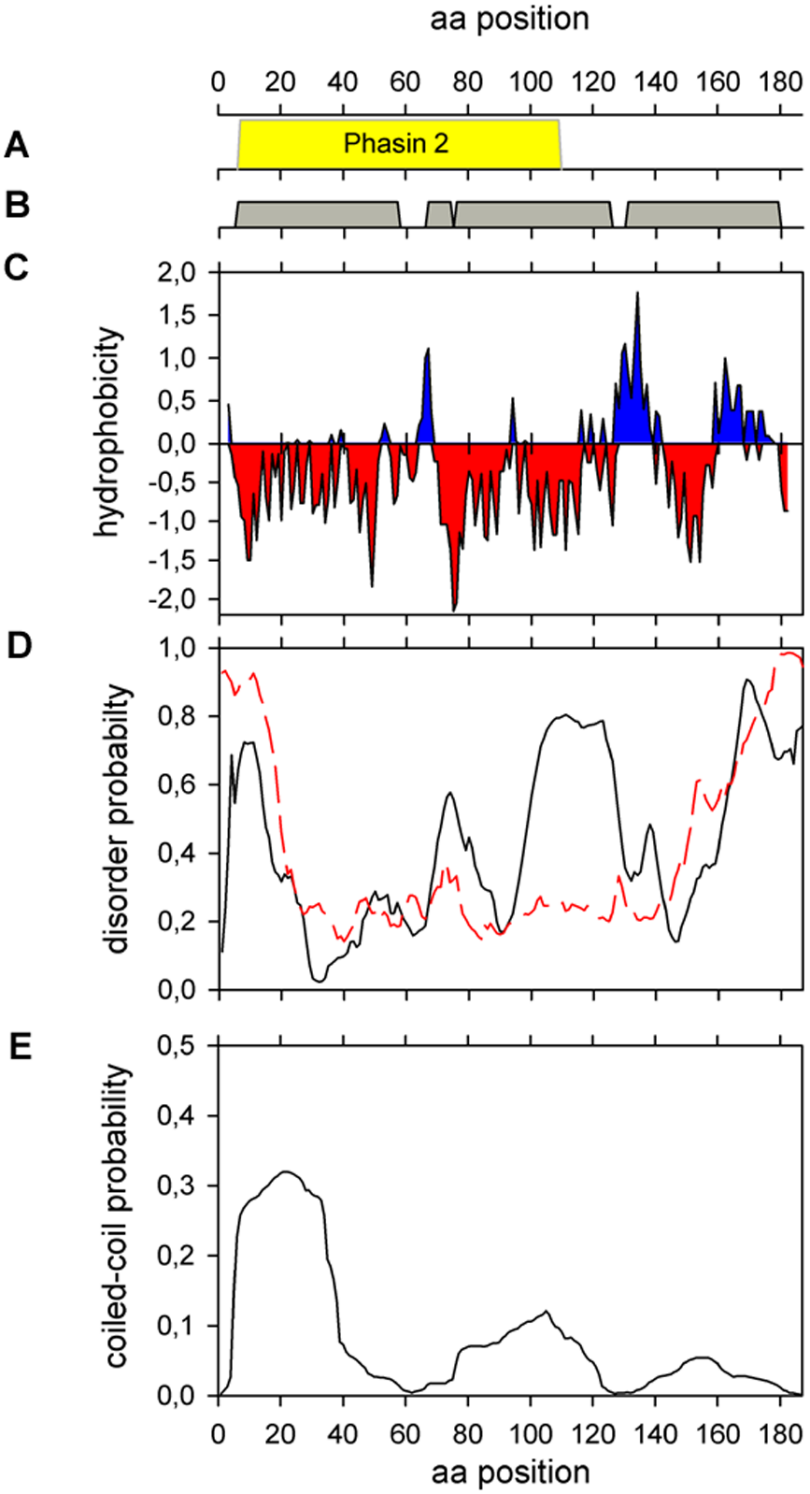
*In silico* analysis of PhaP_Az_ (**A**) **Pfam.** Phasin 2 family (PF09361) conserved sequence (**B**) Phyre2 secondary structure prediction. Helix 1: from L6 to L57, helix 2: from P67 to F74, helix 3: from P76 to N125 and helix 4: from E131 to A177. (**C**) Kyte-Doolittle hydropathicity plot with a window size of 9. (**D**) Protein disorder prediction. Black line: PONDR-VLXT predictor. Red dashed line: PONDR-FIT predictor. (**E**) Probability of coiled- coil regions using MARCOIL.

Many bacteria accumulate polyhydroxyalkanoates (PHAs) as carbon and energy sources. These polymers have thermoplastic properties, are biodegradable, biocompatible and can be produced from a variety of renewable resources, making them an ecologically friendly alternative to traditional petroleum derived plastics [1]. The best known and most common PHA is polyhydroxybutyrate (PHB), composed of 4 C monomers. PHAs are stored intracellularly in granules that are surrounded by a layer of phospholipids and several granule associated proteins. Among these proteins are PHA synthases, PHA depolymerases and a group of proteins known as phasins [2]. There are many different phasins identified in PHA synthesizing bacteria, such as PhaP1, PhaP2, PhaP3, PhaP4 and PhaP5 from *Ralstonia eutropha* (currently denominated *Cupriavidus necator*) [3] [4] [5], GA14 from *Rhodococcus ruber* [6], PhaP from *Bacillus megaterium* [7], PhaF and PhaI from *Pseudomonas putida* [8], and PhbP from *Azotobacter vinelandii* [9], among many others. Apart from their structural role as part of the PHA granule cover, different functions have been found associated to many phasins. Some of them were observed to possess regulatory activity, such as PhaF of *P. putida* (PhaF_Pp_), that regulates PHA synthesis [8] and plays a role in the intracellular location and distribution of PHA granules [10], and ApdA of *Rhodospirillum rubrum*, that activates PHB depolymerization [11]. PhaP1 of *R. eutropha* [12] has been reported to enhance the specific activity of PHA polymerase *in vivo,* and PhaP from *Aeromonas hydrophila* was observed to affect the transcription of *phaC* [13]. Experiments involving phasin mutants have revealed that these proteins affect the number and size of PHA granules [14], and several studies have reported that phasins promote PHA accumulation in natural PHA producers [3], a phenomenon that has also been observed in recombinant *Escherichia coli* [15] [16]. This effect has been attributed to several possible causes, ranging from the activation of genes and/or enzymes involved in PHA synthesis, to acting as a barrier between the polymer and other cellular components, thus avoiding deleterious effects associated to PHA production [17] [3]. In experiments designed to analyze the stress that PHB accumulation caused in recombinant *E. coli*, PhaP of *Azotobacter* sp. FA8 (PhaP_Az_) has been observed to promote growth not only in the cells accumulating PHB; but also in cells that did not carry PHB synthesizing genes. As a result of this unexpected observation, further studies were performed, revealing that PhaP_Az_ had a protective effect in *E. coli* against heat shock and oxidative stress [18]. Phasins have been used in several biotechnological applications, such as the construction of fusion proteins to facilitate protein purification [19] [20]. The multiple roles associated to the different phasins are intriguing, and despite their remarkable functional diversity and their biotecnological applications, the structure of these proteins has not been analyzed except in very few studies. Although phasins do not constitute a highly conserved group of proteins, and early reports indicated that the degree of conservation among them was very low, several protein motifs have been defined using the great number of phasins that have been already described. Considering the Pfam database [21], there are four phasin related families: PF09602 (PhaP_Bmeg), PF09650 (PHA_gran_rgn), PF05597 (Phasin), and PF09361 (Phasin_2). The first corresponds to phasins found primarily in *Bacillus* species such as *B. megaterium*, while the second contains a diverse group of mostly uncharacterized proteins belonging to different Gammaproteobacteria. PF05597 includes 263 sequences belonging to 196 species, most of which are related to *Pseudomonas*, such as PhaF_Pp_. This multifunctional protein, that contains two distinct domains, has been recently characterized in detail using structural prediction programs and biophysical methods, and was proposed to belong to a new family of intrinsically disordered proteins [22]. The last family, PF09361 (Phasin_2), is the most numerous one, as it includes 952 sequences found in 501 species. All phasins from different strains of *Azotobacter,* including PhaP_Az_ (Fig. 1A), belong to this family, that also includes the most studied phasin, PhaP1 from *R.eutropha* (PhaP1_Re_). Structural prediction [23] and mutant analysis revealed that PhaP1_Re_ does not contain a clear hydrophobic domain, and that the whole protein is involved in the interaction with the polymer granules [17], but detailed structural studies have not been performed on proteins belonging to this family. The aim of the present work was to perform a thorough biophysical structural analysis of PhaP_Az_.

## Materials and Methods

### Protein Expression and Purification

Primers phaPup (5′ CATGGGATCCCGTAATGGCTTTTTTTGATC 3′) and phaPlow (5′ TCGAAGCTTGCCGTCAGGCAGTCTT 3′) were used to obtain a 591-bp amplification fragment using plasmid pRX23, containing the *phb* gene cluster from *Azotobacter* sp. FA8, as template. The amplification fragment was cut with HindIII and BamHI and ligated into vector pQE31 (Qiagen). The resulting plasmid, pQP2, confers resistance to ampicillin and expresses *phaP*_Az_ from a promoter operator element consisting of the phage T5 promoter and two *lac* operator sequences. The insert was cloned in such a way that the amino-terminal end of the *phaP*_Az_ gene product is fused to six histidine residues, resulting in a fusion protein. The phasin protein was expressed in strain M15/pREP4 (Qiagen) transformed with pQP2. LB medium supplemented with 1 mM IPTG (isopropyl-β-D-thiogalactopyranoside) was used. Cultures were grown at 37°C and shaken at 250 rpm. After 4 hours of induction, cells were harvested and disrupted by sonication. PhaP was obtained in the soluble fraction after centrifugation at 10,000 g for 20 minutes at 4°C. The protein was purified by immobilized-metal affinity chromatography (IMAC) using a 5 mL His Trap HP column (GE Healthcare), followed by size exclusion chromatography using a Superdex 75 Prep Grade column (GE Healthcare).

The protein was concentrated using a Vivaspin 2 centrifugal concentrator 10 kDa MWCO (Viva Products) and protein concentration was determined spectrophotometrically using a molar extinction coefficient at 280 nm of 4470 M^−1 ^cm^−1^, calculated using the Expasy ProtParam tool.

Mass spectrometric data were obtained using a MALDI-TOF-TOF spectrometer, Ultraflex II (Bruker).

### *In silico* prediction

The secondary structure of the protein was predicted *in silico* using the Phyre2 server, available online (http://www.sbg.bio.ic.ac.uk/phyre2/html/page.cgi?id=index).

We employed the K2D3 algorithm (http://k2d3.ogic.ca/) to deconvolute the far-UV CD spectrum in order to obtain experimental secondary structure contributions.

Hydrophobicity of the protein was evaluated using the Kyte-Doolittle hydropathicity plot (http://gcat.davidson.edu/rakarnik/kyte-doolittle.htm) with a window size of 9.

The Disprot Database of Protein Disorder (http://www.disprot.org/predictors.php) [24] was used to predict disordered/unstructured regions. The predictors used were DisEMBL (http://dis.embl.de) [25] PONDR-FIT and PONDR-VLXT, that additionally predicts Molecular Recognition Features (*MoRFs*), short regions experimentally known to be disordered that become structured when they are co-crystallized with other proteins [26]. We arbitrarily selected those sequences with a disorder disposition higher than 0.5.

Pepwheel (http://emboss.bioinformatics.nl/cgi-bin/emboss/pepwheel) was used to detect potential amphipathic helices.

MARCOIL(http://toolkit.tuebingen.mpg.de/marcoil) was used to predict coiled-coil regions [27] and DrawCoil (http://www.grigoryanlab.org/drawcoil/) was used to create a helical wheel diagram for the postulated coiled-coil tetramer.

### Circular Dichroism Spectroscopy (CD)

CD experiments were carried out in a Jasco J-810 spectropolarimeter equipped with a Peltier PTC-423S system. Isothermal wavelength spectra were acquired at a scan speed of 100 nm/min with a response time of 4 seconds and averaged over at least 6 scans at 25°C.

CD experiments were performed in a buffer containing 20 mM sodium phosphate, 50 mM NaCl pH = 7.3, 1 mM DTT. Increasing concentrations of sodium oleate or 2, 2, 2-trifluoroethanol (TFE) were employed to perform solvent secondary structure stabilization experiments.

### Size Exclusion Chromatography (SEC)

SEC experiments were carried out on an analytical Superdex 200 column (GE Healthcare). The column was calibrated with Thyroglobuline (669 kDa), Aldolase (144 kDa), Bovine Serum albumine (67 kDa) and Myoglobin (17 kDa). The void volume (V_o_) and total volume (V_t_) were determined by loading Blue Dextran and acetone, respectively. The elution buffer contained 20 mM sodium phosphate, 50 mM NaCl pH = 7.3, 1 mM DTT, and the protein concentration was 25 µM. For size exclusion chromatography experiments in denaturing conditions, a buffer containing 20 mM sodium phosphate pH 7.4, 150 mM NaCl, 1 mM DTT, and 6 M GdmCl was used. Protein (25 µM) was treated with 6 M GdmCl and incubated overnight for chemical denaturation.

### Dynamic Light Scattering (DLS)

DLS measurements were carried out on a Zetasizer Nano S DLS device from Malvern Instruments (Malvern). Measurements were performed in 20 mM sodium phosphate pH 7.3 containing 1 mM DTT. PhaP was filtrated with Ultrafree-MC microcentrifuge filters 0.22 mm (Millipore) before measurements were done. Protein concentration was 10 µM. The temperature was maintained at 25°C by a Peltier control system. Results were processed employing the software package included in the equipment.

### Static Light Scattering (SLS)

The average molecular weight of the oligomer was determined by SLS using a precision detector PD2010 light scattering instrument connected in tandem to a high-performance liquid chromatography system and a LKB 2142 differential refractometer. The 90° light scattering and refractive index signals of the eluting material were recorded on a PC computer and analyzed with Discovery32 software supplied by precision detectors. SEC runs were carried out on an analytic Superdex 200 column (GE Healthcare). The protein concentration used in the run was 25 µM and the elution buffer contained 20 mM sodium phosphate pH 7.4, 150 mM NaCl, and 1 mM DTT.

### Thermal and chemical unfolding

Thermal unfolding was followed by molar ellipticity changes at 222 nm employing a heating rate of 2°C min^−1^. The protein concentrations tested were 3 and 1.5 µM. CD spectra at different temperature were performed employing the same conditions described above.

Chemical unfolding of PhaP_Az_ was monitored by CD and fluorescence spectroscopy. The protein was incubated overnight with increasing concentrations of guanidine chloride (GdmCl) in a range from 0 to 5.8 M. The protein concentration tested was 6.8 µM.

Fluorescence emission spectra for 8-anilino, 1-naphtalene sulfonate (ANS) binding to the protein overnight incubated with different concentrations of GdmCl were recorded on an Aminco-Bowman spectrofluorometer with an excitation wavelength of 370 nm and 4 nm band-pass. Samples were incubated with 100 µM ANS (final concentration) at 25°C for 2 h to ensure equilibrium conditions.

## Results and Discussion

### Primary structure of PhaP_AZ_ and comparison to other phasins

As the main role of phasins is to interact with PHA granules, these proteins are usually expected to possess hydrophobic regions. GA14 of *R. ruber,* one of the first granule associated proteins to be characterized, was proposed to interact with PHAs through hydrophobic domains [6]. A Kyte-Doolittle hydropathicity plot [28] of PhaP_Az_ revealed alternative hydrophilic/hydrophobic regions in the C-terminal end of the protein, while strong negative peaks spread throughout the rest of the protein could indicate possible exposed regions (Fig. 1C). Comparison of hydrophobicity plots of PhaP_Az_ with PhaP1 from *R.eutropha* (PhaP1_Re_), that belongs to the same phasin protein family, showed that minima and maxima occur in similar regions of the proteins, especially at the C-terminus (data not shown). None of the two phasins analyzed contained clearly defined hydrophobic regions. A detailed analysis showed that PhaP_Az_ is slightly less hydrophobic than PhaP1_Re_. Other phasins, like the 20 kDa PhaP from *B. megaterium* [7], are very hydrophilic. PhaF_Pp_, a representative of the other family of well characterized phasins, is less hydrophobic than PhaP1_Re_ and GA14, and it lacks a hydrophobic core [22]. While experimental evidence indicated that the hydrophobic domains described in GA14 are essential for binding to PHA granules [6], analysis of PhaP_Re_ mutants revealed that the whole protein is involved in this process, and that there is not one particular region responsible for interaction with the granules [17]. Analysis of the aminoacid sequence of GA14 revealed that it lacks characteristic phasin conserved domains, so the interaction with PHA granules is probably different to phasins belonging to the two main families.

### Secondary structure characterization

In order to start analyzing the secondary structure of PhaP_Az_ we used the Phyre2 server [29]. This approach predicted a high prevalence (87%) of α-helix secondary structure, consisting of four helices: one from L6 to L57, a shorter one from P62 to F73, another from P75 to N125 and the fourth from E131 to A177 (Fig. 1B). These results are in accordance with previous predictions for other phasins, such as those from several species of *Ralstonia*, with around 90% of α-helix secondary structure, and has been proposed to be a general characteristic of these proteins [17].

**Figure 2:**
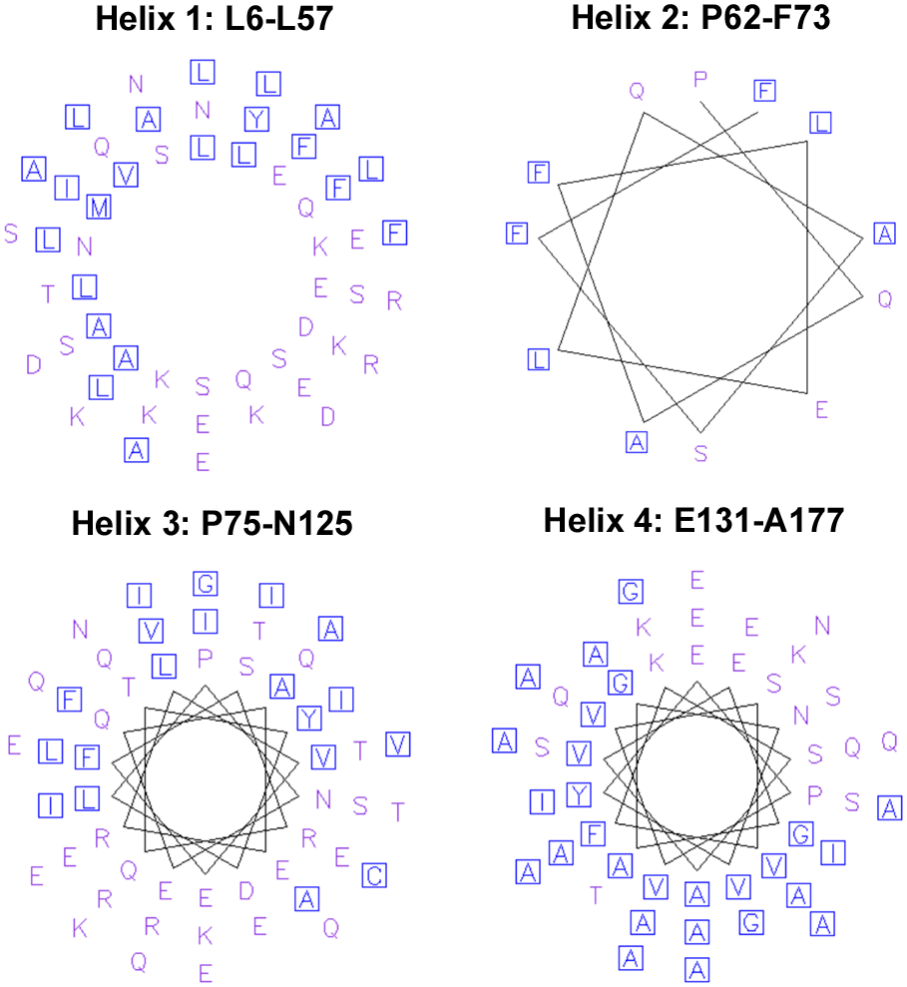
PhaP_Az_ Helical Wheel Diagram. The representation was performed using Pepwheel (http://emboss.bioinformatics.nl/cgi-bin/emboss/pepwheel) that detects potencial amphipathic helices. Residues ACFGILMVWY (polar) are marked as blue squares and all other residues are unmarked.

To further investigate if these predicted helices have amphipathic characteristics that could explain the interactions of PhaP_Az_ with PHA granules, we represented these helices using Pepwheel (http://emboss.bioinformatics.nl/cgi-bin/emboss/pepwheel). (Fig. 2). This representation showed that the first helix (L6 to L57) and the fourth one (E131 to A177) seem to have clearly defined polar and non-polar sides. These amphipathic helices could be involved in the simultaneous interaction of phasins with PHA and an aqueous environment, and also in protein-protein interactions.

To obtain experimental structure information of PhaP_Az_, it was expressed and purified as described in materials and methods. The protein was highly soluble and could be readily purified from the soluble fraction to near homogeneity as judged by SDS-PAGE, that revealed a band of 21 kDa (data not shown). Protein molecular weight and aminoacid sequence were confirmed by MALDI- TOF-TOF.

**Figure 3:**
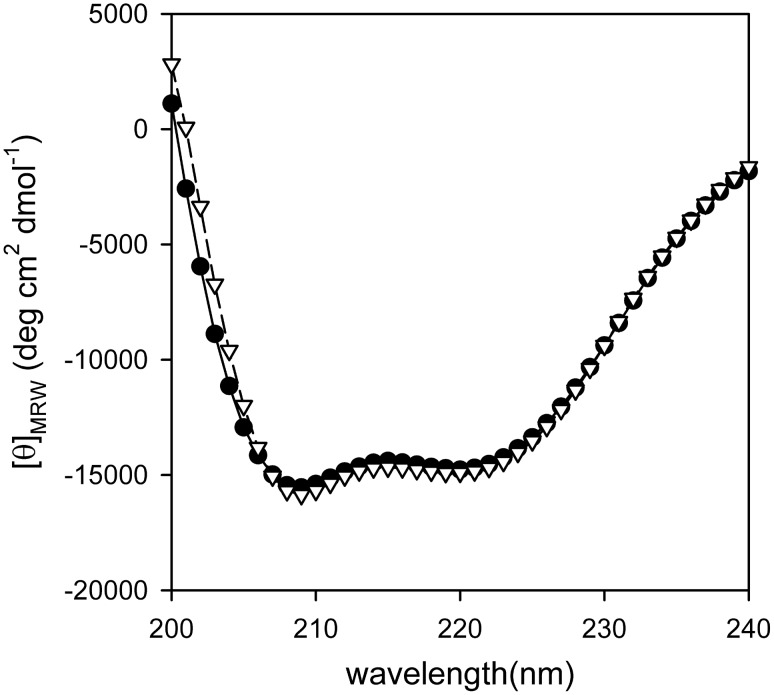
Experimental PhaP_Az_ secondary strucure. Far-UV CD spectrum of PhaP_Az_ 3 µM in sodium phosphate buffer (20 mM, 50 mM NaCl, pH 7.3, 1 mM DTT) (•). Far-UV CD spectrum of PhaP_Az_ predicted by the K2D3 algorithm (∇).

Circular dichroism (CD) was used to obtain experimental information of the secondary structure of the protein. The far-UV CD spectrum of PhaP_Az_ showed two characteristic negative bands at 208 and 222 nm, indicating a typical α-helix fold (Fig. 3). We employed the K2D3 algorithm to deconvolute the far-UV CD spectrum in order to obtain secondary structure contributions that revealed a 38% of α-helix.

As the experimental value was much lower than the 87% predicted by *in silico* analysis, some methods contained in the Disprot Database of Protein Disorder [24] were used to investigate if PhaP_Az_ could have disordered/unstructured regions, as described for other phasins [22]. Using DisEMBL [25], and selecting the predicted regions with non assigned electron densities that most often reflect intrinsic disorder, three segments where predicted to be disordered: M1-K14, E149-S159, and A169-A187. Similar results were found with PONDR-FIT [26], that predicted two disordered regions at the N- terminal (M1-A19) and C- terminal (Q152-A187) ends. PONDR-VLXT, that predicts Molecular Recognition Features (*MoRFs*), found an additional disordered region between V100-A128 (Fig. 1D). From these data we estimated that around 40/45% of PhaP_Az_ could be disordered when it is not binding any target, and this value would decrease to 23/30% when interacting with other molecules. The amount of disorder predicted in the absence of target is compatible with the α-helix content obtained experimentally, and could explain the discrepancies with the secondary structure prediction. Disordered regions have been found in many different kinds of proteins involved in diverse functions, including histones, proteins that control the cell division cycle, and chaperones [30]. These disordered regions have been predicted in a great number of phasins, and have been previously experimentally observed in PhaF_Pp_. This protein has been shown to have an intrinsically unstructured domain in its DNA binding C-terminal domain and an additional disordered region in its N-terminal domain, that was proposed to interact with the granule [22]. Disordered regions are thus apparently a common feature of phasins, suggesting that they could contribute to the functional diversity of these proteins.

### Influence of the environment on PhaP_AZ_ secondary structure

**Figure 4:**
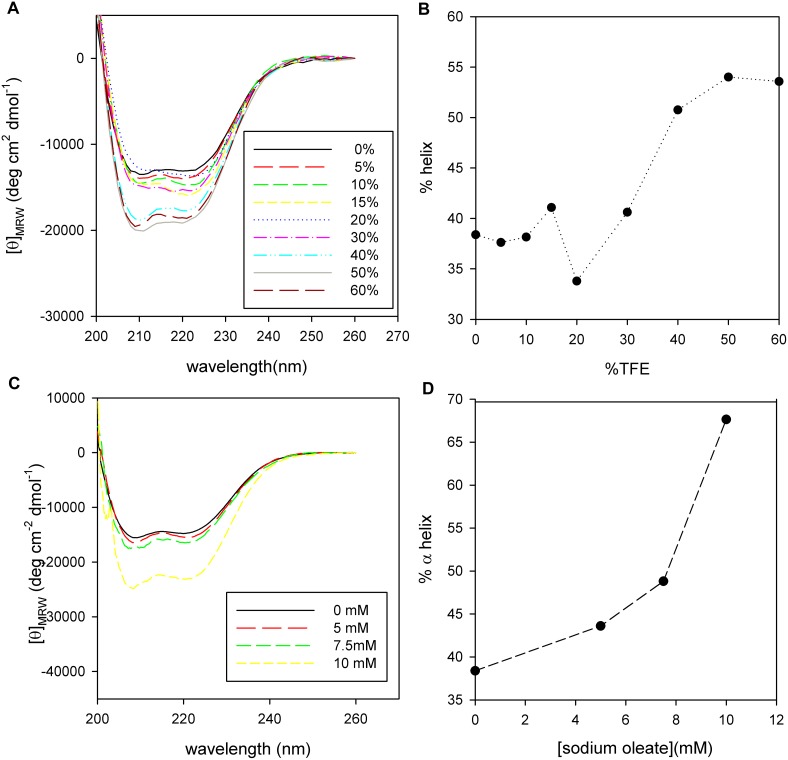
PhaP_Az_ secondary structure in different environments. CD Spectra of PhaP_Az_ (3 µM) in sodium phosphate buffer (20 mM, NaCl 50 mM, pH 7.3, 1 mM DTT) (**A**) at different TFE concentration (from 0 to 60%) or (**C**) at different sodium oleate concentrations (0 to 10 mM). α-helix content, calculated using K2D3 algorithm, as a function of TFE (B) or sodium oleate (D).

To investigate the possibility that the the proportion of α-helix in PhaP_Az_, is affected by the environment, the CD spectra of the protein were analyzed in the presence of different amounts of TFE, a solvent that generally stabilizes preexisting α-helical populations in peptides and proteins [31] [32]. The proportion of α-helix (calculated using K2D3 to deconvolute the far-UV CD spectra) was increased by addition of TFE, reaching a saturation value of 55% of the aminoacids in α-helix conformation with 50% TFE (Fig. 4A and B).

If observed carefully, it can be seen that although the relationship between the two characteristic minima (222 nm/208 nm) remains close to 1, indicating that the protein is predominantly in α-helix conformation, this ratio varies in a non-monotonous way. This behavior shows that there is not a two-state transition, revealing a more complex secondary structure reorganization. This suggests some degree of flexibility that could enable structural changes to be induced by the environment.

As phasins are normally found in the lipid surface of PHA granules, the possibility that the structure of PhaP_Az_ could be affected by a lipidic environment was further analyzed using sodium oleate, a common coating component used as a hydrophobic mimic of PHA for *in vitro* analysis [22]. The degree of α-helix increased sharply in the presence of oleate, reaching a value of 68% of the aminoacids in α-helix conformation with 10 mM of oleate (Fig. 4C and D). It was not possible to see if it would reach a plateau region due to oleate solubility limits. The behavior of PhaP_Az_ in the presence of oleate indicates that this protein is able to change its structure in a hydrophobic environment and suggests that it could reach the α-helix amount predicted by *in silico* analysis when interacting with a hydrophobic moiety such as PHA.

### Tertiary and quaternary structure characterization

**Figure 5:**
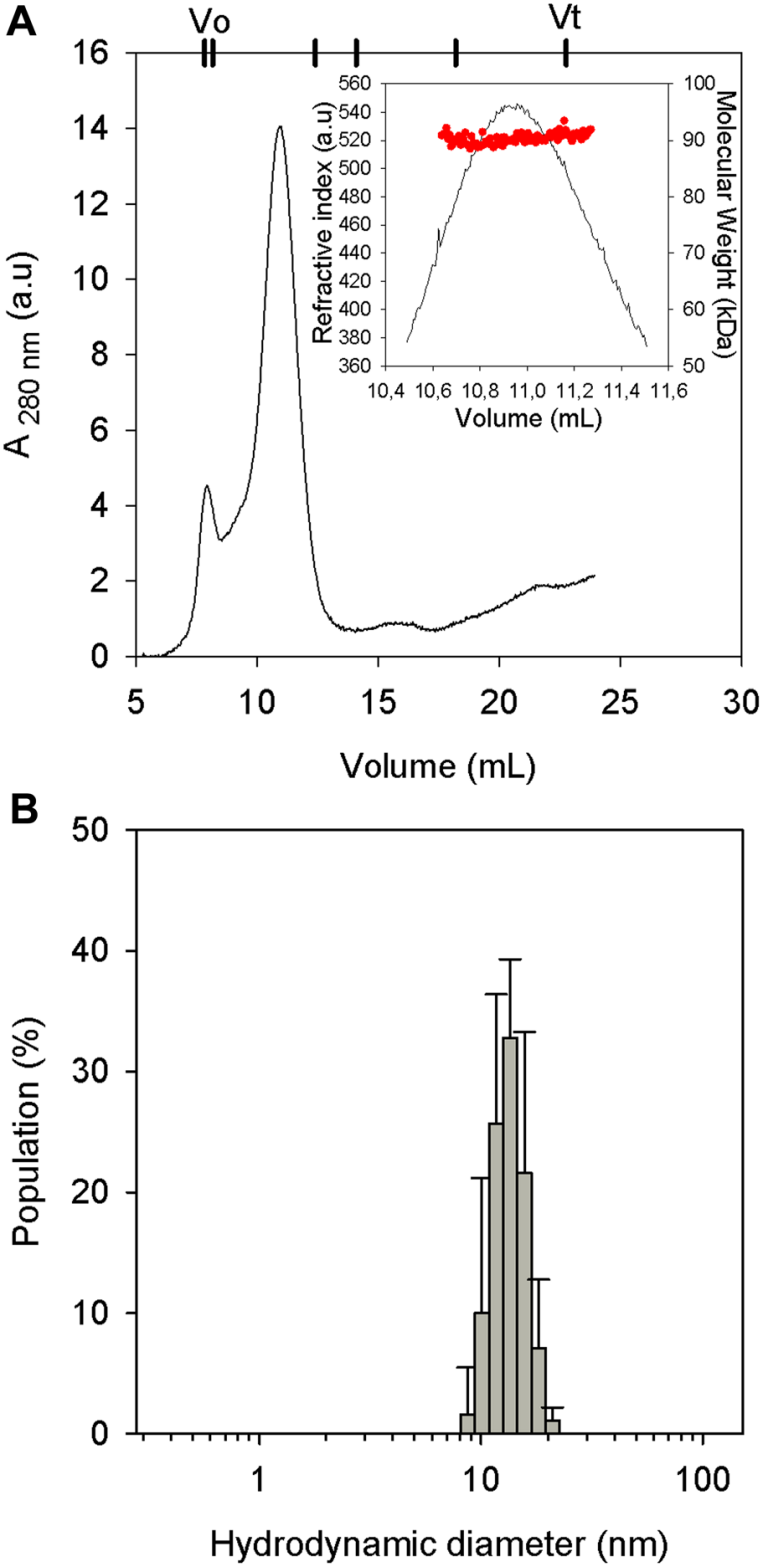
PhaP_Az_ molecular weight estimation. (**A**) Elution profile of 25 µM PhaP_Az_ by SEC using an analytical Superdex 200 column. The elution volumes corresponding to Blue dextran (Vo),Thyroglobuline (669 kDa), Aldolase (144 kDa), Bovine Serum albumine (67 kDa), Myoglobin (17 kDa) and acetone (Vt are indicated at the top. Inset: SLS determination. (**B**) DLS measurements of 10 µM PhaP_Az_ sample.

PhaP_Az_ is a protein of 21 kDa, but it eluted close to the void volume when using a Superdex 75 column in a size exclusion chromatography experiment (data not shown). This elution volume corresponds to a globular protein of at least 75 kDa. This hydrodynamic behavior was confirmed by a DLS experiment in which a diameter of 12 nm was obtained (Fig. 5B), while a globular monomer of 21 kDa would be expected to have a hydrodynamic diameter of 4.4 nm. This hydrodynamic diameter is also bigger than what would be expected for an unfolded (8.4 nm) or natively unfolded monomer of this size (6.4–7.6 nm) [33]. Even though we predicted disordered regions in PhaP_Az_ and these hydrodynamic techniques use globular protein models to calculate diameters and molecular masses, the results obtained suggested that the protein was not monomeric in these conditions. When an analytical Superdex 200 filtration column was employed, the elution volume of PhaP_Az_ corresponded to a globular protein of approximately 200 kDa (Fig. 5A). This molecular weight corresponds to 9 monomers. However, taking into account that the secondary structure characterization revealed disordered regions, the oligomer would be expected to have a lower molecularity. In order to more precisely determine it, SLS experiments were performed, indicating an average molecular weight of 90 kDa (Fig. 5A, inset). Taking into account that the expected weight for a PhaP_Az_ monomer is 21 kDa, this molecular weight is compatible with a tetramer. This result is in accordance with the oligomerization state of trimers [17] or tetramers [34],[22] postulated for other phasins in the literature.

**Figure 6:**
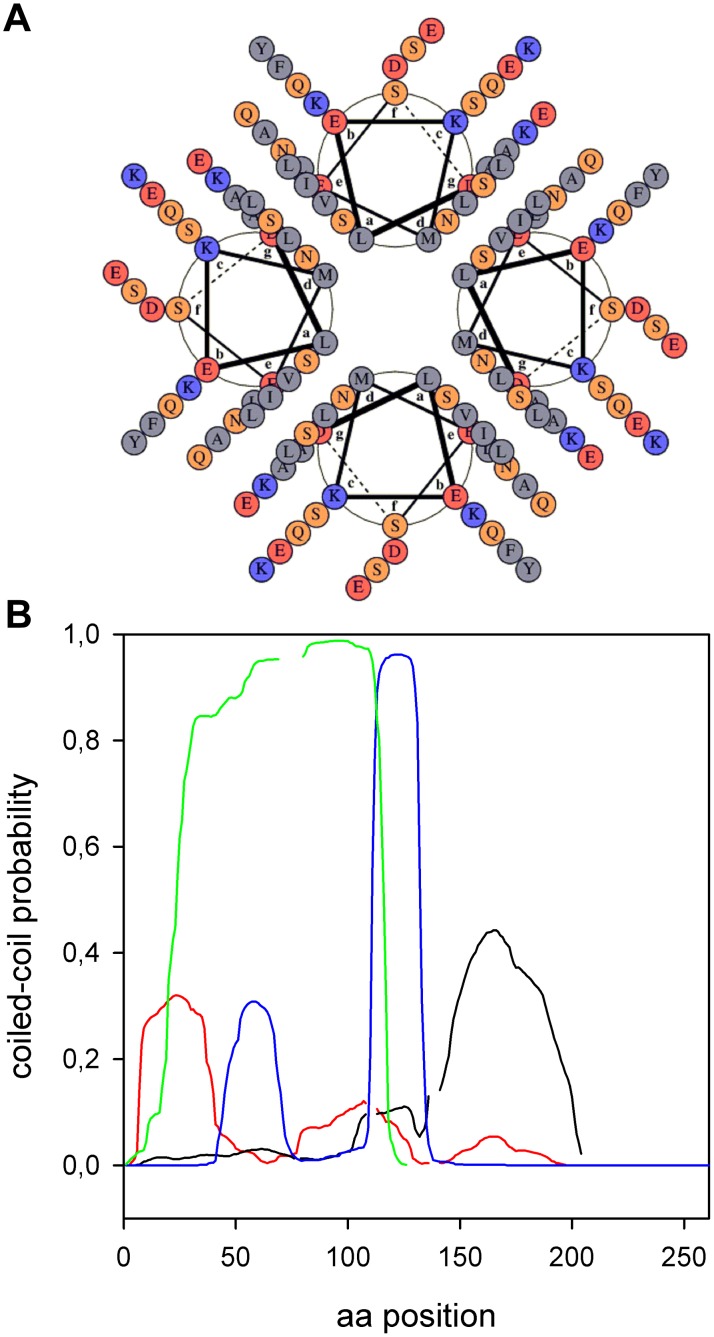
Phasins coiled-coil regions. (**A**) Helical-wheel representation of the predicted coiled-coil interacting as a parallel tetramer. The helices correspond to the aminoacid sequence from D5 to Q38 with a maximum probability of 32% of coiled-coil interaction (MARCOIL prediction), the scheme was done using DRAWCOIL. (**B**) Comparison of coiled- coil regions from different phasins (MARCOIL prediction). Red line: PhaP_Az_, black line: PhaP1_Re_, blue line: PhaF_Pp_ and green line: PhaP from *Aeromonas hydrophila*.

It was previously described that some phasins have a high probability (>75%) while others have an intermediate probability (25 to 75%) of containing coiled-coil sequences [22]. Using MARCOIL [27] to find coiled-coil regions, PhaP_Az_ was determined to be part of the second group, as it has a region between D5 and Q38 with a maximum probability of 32% of coiled-coil interaction that could be a homo-oligomerization domain, at least in the absence of other targets. Fig. 6A shows a Helical-wheel representation of the predicted coiled-coil interacting as a parallel tetramer, using DRAWCOIL.

It is interesting to mention that the coiled-coil regions for different phasins are located in different positions along their aminoacid sequences (Fig. 6B). PhaF_Pp_ has a coiled-coil region with a high probability between its two domains, but it also has a moderate probability one in the middle of its N-terminal domain. PhaP1_Re_, that has a hydropathicity pattern very similar to PhaP_Az_, also has a coiled-coil region, with a similar probability of occurrence, but in the opposite end of the sequence. PhaP from *Aeromonas hydrophila,* another phasin included in the Phasin 2 family, has a high coiled-coil probability along its entire sequence.

As it was mentioned above (Fig. 4A and B) when PhaP_Az_ was incubated with increasing amounts of TFE, the α-helix content increased in a biphasic way, and the ratio between the two characteristic minima (222 nm/208 nm) presented a maximum at intermediate TFE concentrations. This ratio initially began close to 1, then it started to increase with increasing TFE concentrations until it reached a maximum around 20–25% TFE, and finally ended in a value lower than 1. It has been previously reported that for coiled-coil interacting peptides without any other secondary structure than α-helix, the coiled-coil structure presents a ratio equal or greater than 1, and a ratio close to 0.9 represents non interacting α-helices [35] [36]. Taking this into account, the observed behavior can be interpreted in the following way: in an aqueous environment PhaP_Az_ could be forming a coiled-coil interacting oligomer that is stabilized by the addition of low amounts of TFE, while higher amounts of TFE could induce the conversion to a more rigid single stranded α-helix. In experiments in which oleate was added, increasing amounts of this hydrophobic compound produced a decrease in the 222 nm/208 nm ratio, indicating that the increment in α-helix content could be accompanied by a stabilization of single stranded α-helix. Careful analysis of PhaP_Az_ CD spectra in aqueous buffer solution using different protein concentrations revealed that the 222 nm/208 nm ratio was slightly concentration dependent, as expected for non-covalently bonded, coiled-coil interacting α-helices (not shown) [37].

### Thermal and chemical unfolding

**Figure 7:**
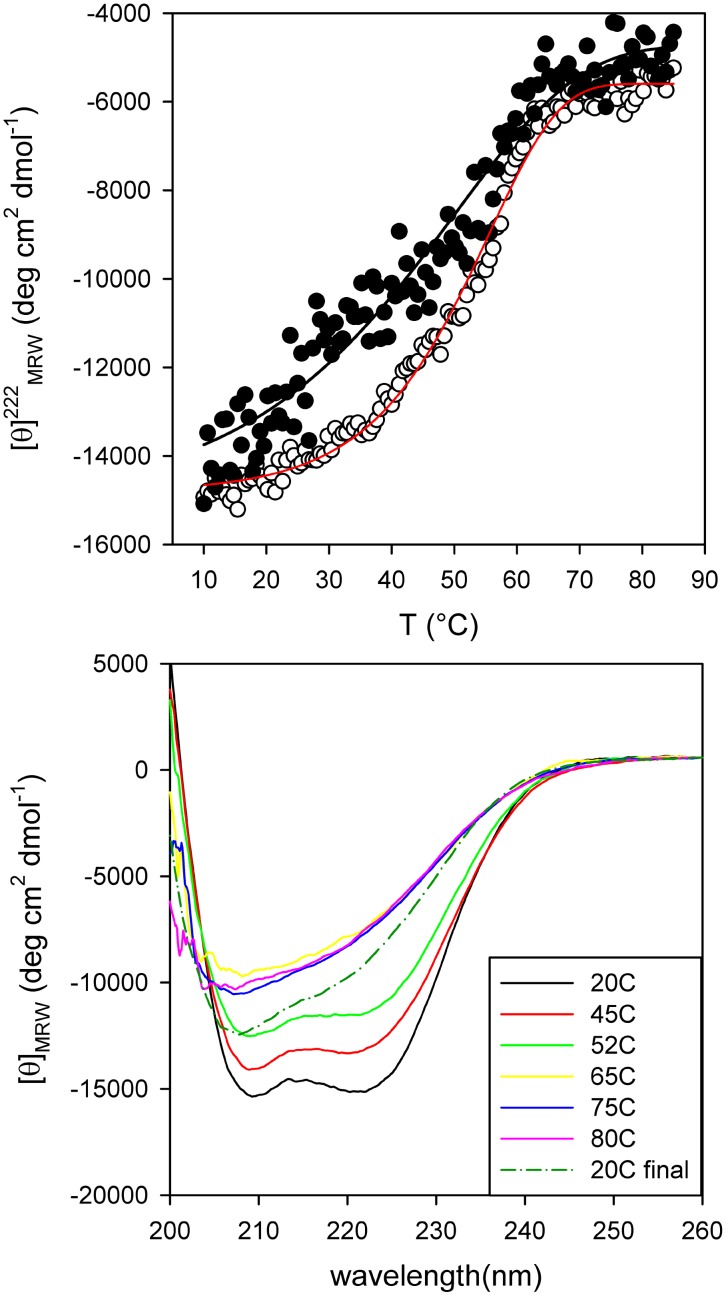
PhaP_Az_ thermal stability. (**A**) Denaturation was followed by measuring the ellipticity change in the far-UV region at 222 nm. (○) PhaP_Az_ 3 µM (apparent Tm = 55°C) (•) PhaP_Az_ 1.5 µM. (**B**) Far-UV CD Spectra of PhaP_Az_ (3 µM) in 20 mM sodium phosphate buffer pH 7.3 containing NaCl 50 mM and 1 mM DTT at different temperatures (from 20°C to 80°C). Recovered spectra were registered upon immediate cooling down the scanned sample and after a 10 min waiting period at 20°C.

The thermal denaturation of PhaP_Az_ was monitored by CD. At 222 nm, where the largest change in ellipticity took place, a sharp symmetric transition could be observed (Fig. 7A). When using 3 µM protein, the cooperative transition started at 30°C, showed an apparent Tm of 55°C, and was completed at 65°C. The thermal unfolding process was not reversible, as above 65°C the protein tended to aggregate, and the native spectrum was not recovered completely upon cooling (Fig. 7B). This aggregation was likely caused by residual secondary structures at the end of the transition. The isodichroic point observed before protein aggregation (∼205 nm) indicates that the complete unfolding (between the folded oligomer and the unfolded state) was a two state process. The lower Tm and the decrease in cooperativity observed at lower protein concentrations are consistent with the monomerization process occurring during unfolding (Fig. 7A).

**Figure 8:**
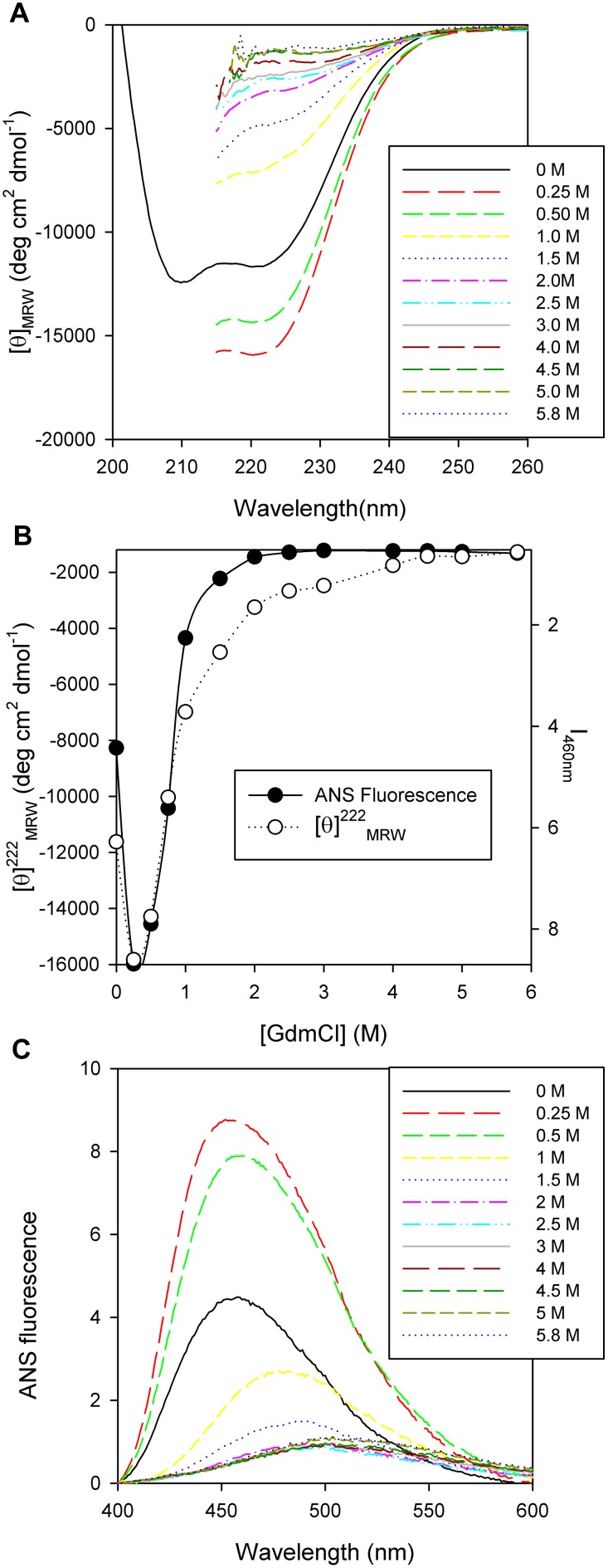
PhaP_Az_ secondary and tertiary/quaternary structural change. (**A**) Far-UV CD Spectra of PhaP_Az_ (7 µM) in 20 mM sodium phosphate buffer pH 7.3 containing 50mM NaCl and 1 mM DTT, at different GdmCl concentrations in the range of 0 to 5.8 M. (**B**) PhaP_Az_ chemical unfolding monitored by fluorescence spectroscopy and CD: (•) ANS fluorescence as a function of GdmCl (○) Molar ellipticity at 222 nm as a function of GdmCl concentration. (**C**) Fluorescence spectra of ANS binding to PhaP_Az_ (7 µM) treated with different concentrations of GdmCl in the range of 0 to 5.8 M.

PhaP_Az_ unfolding was characterized using GdmCl as a chemical denaturation agent. Secondary structure changes, monitored by CD, did not fit to a two state model, indicating the existence of at least an intermediate species (Fig. 8 A and B). A first transition was observed at 0.25 M GdmCl, and the unfolding process occurred between 0.25 and 5.8 M GdmCl. At 0.25 M GdmCl, the far UV- CD spectrum showed an increment of negative ellipticity that corresponds to an increase in secondary structure. This could be interpreted as a compactness of the oligomer due to a more globular-like behavior. The molar ellipticity did not change from 4.5 to 5.8 M GdmCl indicating that the unfolded state was reached at that point.

As the protein has no tryptophan residues that could be used to sense changes in the tertiary/quaternary structure, ANS fluorescence experiments were performed to further analyze these changes during chemical unfolding. The ANS dye is known to present different maximum emission wavelength positions and fluorescence intensity that reflect its binding to hydrophobic surfaces or different polarity environments [38]. Changes in tertiary/quaternary structure displayed a biphasic behavior, in a similar way as was observed for the secondary structure (Fig. 8B and C). The compacted species formed at 0.25 M GdmCl presented a dramatic fluorescence increment, accompanied by a spectral blue shift that reflects a more hydrophobic ANS environment. As GdmCl concentration increased, the fluorescence emission intensity decreased and the spectra shifted to red, showing a more hydrophilic environment. At the end of the curve, the spectra reached a value of 513 nm, close to the value of 520 nm obtained in the buffer solution, indicating that ANS is almost completely exposed to an aqueous environment. The comparison of CD and ANS measurements showed that while the secondary structure change reached a plateau at 4.5 M GdmCl, the tertiary/quaternary structure change was almost complete at 2 M GdmCl, indicating that after forming the compact intermediate at 0.25 M GdmCl, the protein needed less GdmCl to completely lose its tertiary/quaternary structure (Fig. 8B).

**Figure 9:**
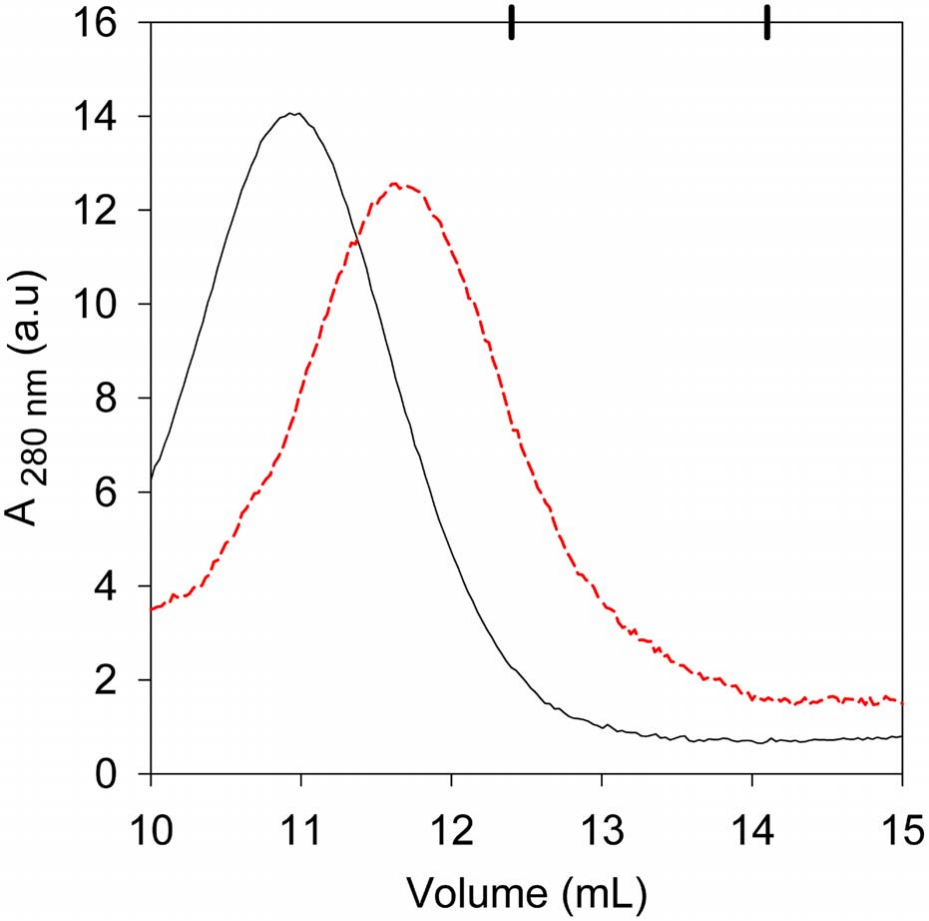
PhaP_Az_ size change after chemical unfolding. Black line: profile elution of native PhaP_Az_ (0 M GdmCl). Red dashed line: profile elution of chemically denatured PhaP_Az_, (6 M GdmCl).

In order to know if the protein presented a size change after unfolding compatible with these results, we performed a Superdex 200 filtration experiment employing a 6 M GdmCl incubated protein sample. As shown in Fig. 9, the unfolded sample eluted at a higher volume than the folded one, indicating that its size got smaller when unfolding. The unfolded protein eluted in a volume that corresponds to a globular protein with a hydrodynamic diameter of 8.9 nm (158 kDa). This value was in good agreement with the expected 8.4 nm hydrodynamic diameter for an unfolded protein of 21 kDa [33]. These results permitted us to conclude that the protein was a monomer at the end of the unfolding curve.

Taking together these results, the chemical unfolding of PhaPAz could be interpreted in the following way: the oligomer structure is first compacted, then an increment in GdmCl concentration produces the loss of tertiary/quaternary structure leading to protein monomerization, and at the highest concentrations the monomer loses its secondary structure.

## Conclusions

Among the proteins associated to PHA granules, phasins constitute a very diverse group, and although they all appear to have a structural role as part of the granule cover, a few that have been studied in detail revealed that they have different additional functions. Phasins have also been used in several biotechnological applications, such as the construction of affinity tags to facilitate protein purification. Several types of phasin families have been distinguished based on sequence similarity, but *in silico* predictions indicate that they apparently share some structural features, such as a preponderant α-helix composition combined with disordered regions. Similar structural characteristics were predicted and experimentally verified for PhaP_Az_. The higher hydrodynamic diameter obtained in DLS measurements and the smaller elution volume in SEC experiments compared with the expected values for a globular protein of 21 kDa, together with the lower α-helix composition in comparison with *in silico* predictions, evidenced the presence of disordered regions in this protein.

All phasins contain coiled-coil interacting regions, so these could be postulated as oligomerization surfaces, but the location and extension of these regions differs greatly, even among phasins belonging to the same family. This suggests that the interaction between monomers and with PHA granules, other phasins and other proteins could differ for different phasins.

Phasins belonging to the different families have been proposed to be trimers [17] or tetramers [34] [22]. Results presented in this work, like the protein size determined by SLS, the size change observed after chemical unfolding, the ellipticity ratio between 222 nm/208 nm and the single sharp melting profile, evidenced that PhaP_Az_ is a coiled-coil tetramer when it is not binding any target. Taking into account the results obtained in the experiments with TFE and oleate, it can be proposed that the molecularity of the protein could change depending on the environment or interactions with other molecules, such as when binding to a target. PhaP_Az_ showed a remarkable capacity to change according to its environment, probably due to its unstructured regions, and this structural flexibility suggests that it could interact with different targets, including other proteins. The results presented in this work contribute to the understanding of the diverse functional properties observed for phasins, and will be useful to develop new biotechnological applications using these remarkable proteins.
